# Changes in fatty acid composition in the giant clam *Tridacna maxima* in response to thermal stress

**DOI:** 10.1242/bio.017921

**Published:** 2016-08-19

**Authors:** Vaimiti Dubousquet, Emmanuelle Gros, Véronique Berteaux-Lecellier, Bruno Viguier, Phila Raharivelomanana, Cédric Bertrand, Gaël J. Lecellier

**Affiliations:** 1 EPHE, PSL Research University, UPVD-CNRS, USR3278 CRIOBE, 98729 Moorea, French Polynesia; 2 University of French Polynesia-Ifremer-ILM-IRD, UMR241 EIO, BP 6570, 98702 Faa'a, Tahiti, French Polynesia; 3 Département de recherche agronomique appliquée, Service du développement rural, BP 100, Papeete, Tahiti 98713, French Polynesia; 4 EPHE, PSL Research University, UPVD-CNRS, USR3278 CRIOBE, 66860 Perpignan, France; 5 Université Paris-Saclay/Versailles-Saint Quentin en Yvelines, 55 Avenue de Paris, 78035 Versailles Cedex, France; 6 Laboratoire d'Excellence “CORAIL”, 58 Avenue Paul Alduy, 66860 Perpignan Cedex, France

**Keywords:** Thermal stress, Fatty acids, Differential expression, Antioxidant, *Symbiodinium*, *Tridacna maxima*

## Abstract

Temperature can modify membrane fluidity and thus affects cellular functions and physiological activities. This study examines lipid remodelling in the marine symbiotic organism, *Tridacna maxima*, during a time series of induced thermal stress, with an emphasis on the morphology of their symbiont *Symbiodinium*. First, we show that the French Polynesian giant clams harbour an important proportion of saturated fatty acids (SFA), which reflects their tropical location. Second, in contrast to most marine organisms, the total lipid content in giant clams remained constant under stress, though some changes in their composition were shown. Third, the stress-induced changes in fatty acid (FA) diversity were accompanied by an upregulation of genes involved in lipids and ROS pathways. Finally, our microscopic analysis revealed that for the giant clam's symbiont, *Symbiodinium,* thermal stress led to two sequential cell death processes. Our data suggests that the degradation of S*ymbiodinium* cells could provide an additional source of energy to *T*. *maxima* in response to heat stress.

## Abstract (precis)

**Summary:** Lipid remodelling according to the gene expression of *Tridacna maxima* and the fate of its symbiont *Symbiodinium* may contribute to its resistance during thermal stress.

## INTRODUCTION

Temperature plays a role in various cellular functions, including the development of protein structure, velocity of chemical and enzymatic reactions, membrane fluidity, and diffusion (Hochachka and Somero, 2002). Because ectotherms depend on environmental conditions for body temperature, fluctuating temperatures affect their physiological activities. To avoid or mitigate the effects of changes in temperature, they develop various adaptive patterns of behaviour (e.g. depending on temperature, the eastern brown snake either basks in the sun or becomes active at night) or they adapt physiologically. One well-known cellular response to increasing temperatures is the expression of heat shock proteins which help reduce the unfolding effect on proteins. Membrane-dependent processes are sensitive to temperature because of the physio-chemical properties of membrane lipids that influence membrane fluidity. Membrane fluidity, under the influence of associated proteins, is reduced with decreasing temperatures and enhanced by increasing temperatures, leading to membrane dysfunction. The usual response to this temperature effect is the remodelling of membrane lipids, known as homeoviscous adaptation (HVA) (Sinensky, 1974). HVA characterises the maintenance of the membrane fluidity through changes in phospholipid head groups, fatty acid (FA) composition and cholesterol content (Crockett and Hazel, 1995). These changes in FA composition primarily affect the saturated (SFA) versus unsaturated fatty acids (UFA) and their chain lengths. For example, with higher temperatures, the membrane FAs become more saturated and/or have longer carbon chains to compensate for the increasing fluidity. The opposite occurs when temperatures are decreased (Hochachka and Somero, 2002). These modifications mainly reflect a shift in the lipid metabolism that is independent of food supply (Brodte et al., 2008; Jangaard et al., 1967).

In symbiotic marine organisms, photosynthetic symbionts, such as the dinoflagellates from the genus *Symbiodinium* found in corals and giant clams, provide photosynthetically fixed carbon to their host, which contributes to their lipid composition (Burriesci et al., 2012; Hawkins and Klumpp, 1995; Ishikura et al., 1999; Johnston et al., 1995; Papina et al., 2003; Treignier et al., 2008). Some of these compounds, such as stearidonic acid (SDA), were shown to be biomarkers of the symbiont (Imbs and Yakovleva, 2012; Kneeland et al. 2013; Papina et al., 2003). Similarly, the host can contribute to the lipid content of its symbionts (Imbs et al*.*, 2014). In corals, this relationship lends to distinct lipid composition, depending on numerous factors such as the species, the seasons, the depth of habitat, the amount of light, clades of *Symbiodinium* and other environmental factors [for review, see Imbs, (2013)]. Under thermal stress, both the coral host and its *Symbiodinium* contribute to the physiological response, changing their FA composition under bleaching conditions or short-term thermal stress. According to the HVA hypothesis, during periods of thermal stress, hard corals generally exhibit a decrease in total lipids in combination with a reduction in polyunsaturated fatty acids (PUFA) content (Bachok et al*.*, 2006; Imbs and Yakovleva, 2012; Tolosa et al*.*, 2011).

However, other interactions between cellular pathways may occur, which also have the capacity to elicit membrane lipid remodelling, such as induced oxidative stress during thermal stress (An et al*.*, 2010; Kraffe et al*.*, 2007), as well as various factors including historical traits (Hazel, 1984; Van Dooremalen et al*.*, 2011; Williams and Somero, 1996), daily variations in the environment (Pernet et al*.*, 2007a), reproduction (Stanley, 2006; Stanley et al*.*, 2009), age (van Dooremalen and Ellers, 2010) and diet (Haubert et al*.*, 2004; Treignier et al*.*, 2008, 2009). Moreover, due to the complexity of such numerous factors involved in lipid content remodelling, lipid compositions are species and/or tissue dependent (Hall et al*.*, 2002; Hazel, 1984; Logue et al*.*, 2000; Pernet et al*.*, 2007b).

*Tridacna maxima* is one of the positive contributor organisms to coral reefs, serving as a shelter for many life forms, providing food to predators and scavengers, and supplying a significant amount of O_2_ to the environment (Neo et al., 2015). Interestingly, observations made after mass bleaching events (Andréfouët et al., 2013; Buck, 2002) have shown that the *Symbiodinium* symbiotic organism is more resistant than corals to heat stress, suggesting that these two symbiotic organisms have different acclimation capacities, and possibly even distinct HVA. As is the case for many marine organisms, this species is rich in a special class PFA (C20:3-n3, C20:4-n6, C22:4-n6) (Johnston et al., 1995; Mostafa and Khalil, 2014). Our study used a stress time-series to better understand how *T. maxima's* complex lipid content remodelling system responds to thermal stress and the mechanics behind this process. The aims of our study were (1) to determine, *in situ*, the lipid composition for *T.* *maxima*; (2) to determine the response of FA composition during thermal stress; together with (3) changes in the expression of genes involved in the lipid pathways and encoding antioxidant enzymes; and (4) to observe the cellular morphology of *Symbiodinium* when thermally stressed.

## RESULTS

### The lipid content at 26°C is equally composed of saturated and unsaturated FA

A two-way ANOVA of all control data (day 0 of stressed tanks and time series of unheated tanks) did not lead to significant differences in the proportions of the FA according to the sampling day (Df=150, *F*=0.913, *P*=0.74). Therefore, the results were combined and used as a control (C0) for subsequent analyses to increase the power of the statistical tests and to include the most comprehensive variance as possible, in the control conditions.

In total, 30 FA constituents were identified (Table 1), 10 saturated (SFA) contributing 50.64% to the total FA, the remaining part being composed of 20 unsaturated FA [13.32% monounsaturated fatty acids (MUFA), 36.04% polyunsaturated fatty acids (PUFA)]. SFA were mainly constituted of 35% C16:0, 7% C18:0 and 4.5% C14:0. The occurrence of two hydroxylated SFA, C16:0,2-OH and C18:0,2-OH, was observed, while a one branched SFA, 7-methyl-6-hexadecenoic acid (C16:1,7-Me) was identified as a minor constituent. The MUFA major constituents were palmitoleic acid (C16:1 n-7) at 6.91% and oleic acid (C18:1 n-9) at 4.93%. PUFA were mainly composed of five components: stearidonic acid (SDA, C18:4 n-3) at 12.31%, docosahexaenoic acid (DHA, C22:6 n-3) at 6.98%, γ-linoleic acid (GLA, C18:3 n-6) at 4.08%, arachidonic acid (ARA, C20:4 n-6) at 3.93% and octadecapentaenoic acid (C18:5 n-3) at 2.26%. The n-3 PUFA (23.62%) was dominant, at levels twice that of the n-6 PUFA (11.97%), and four times that of the n-9 MUFA (6.70%). Concerning cholesterol, its level in controls was found to be at 1.35% of the apolar fraction. One non-methylene interrupted compound was detected (C22:2-7,15) at a low proportion (0.77%).

**Table 1.**
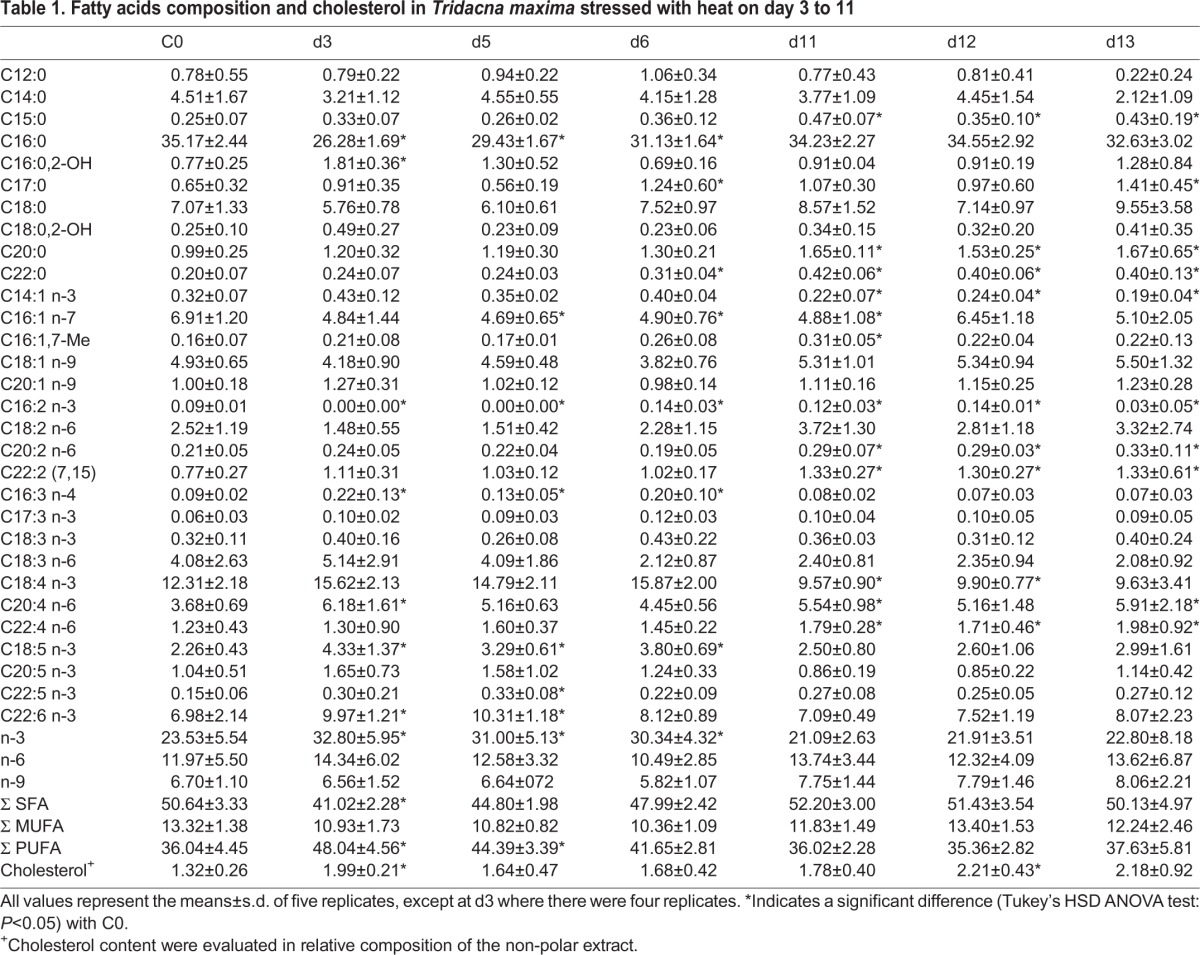
**Fatty acids composition and cholesterol in *Tridacna maxima* stressed with heat on day 3 to 11**

### SFA and PUFA are highly regulated during thermal stress

**Fig. 1.**
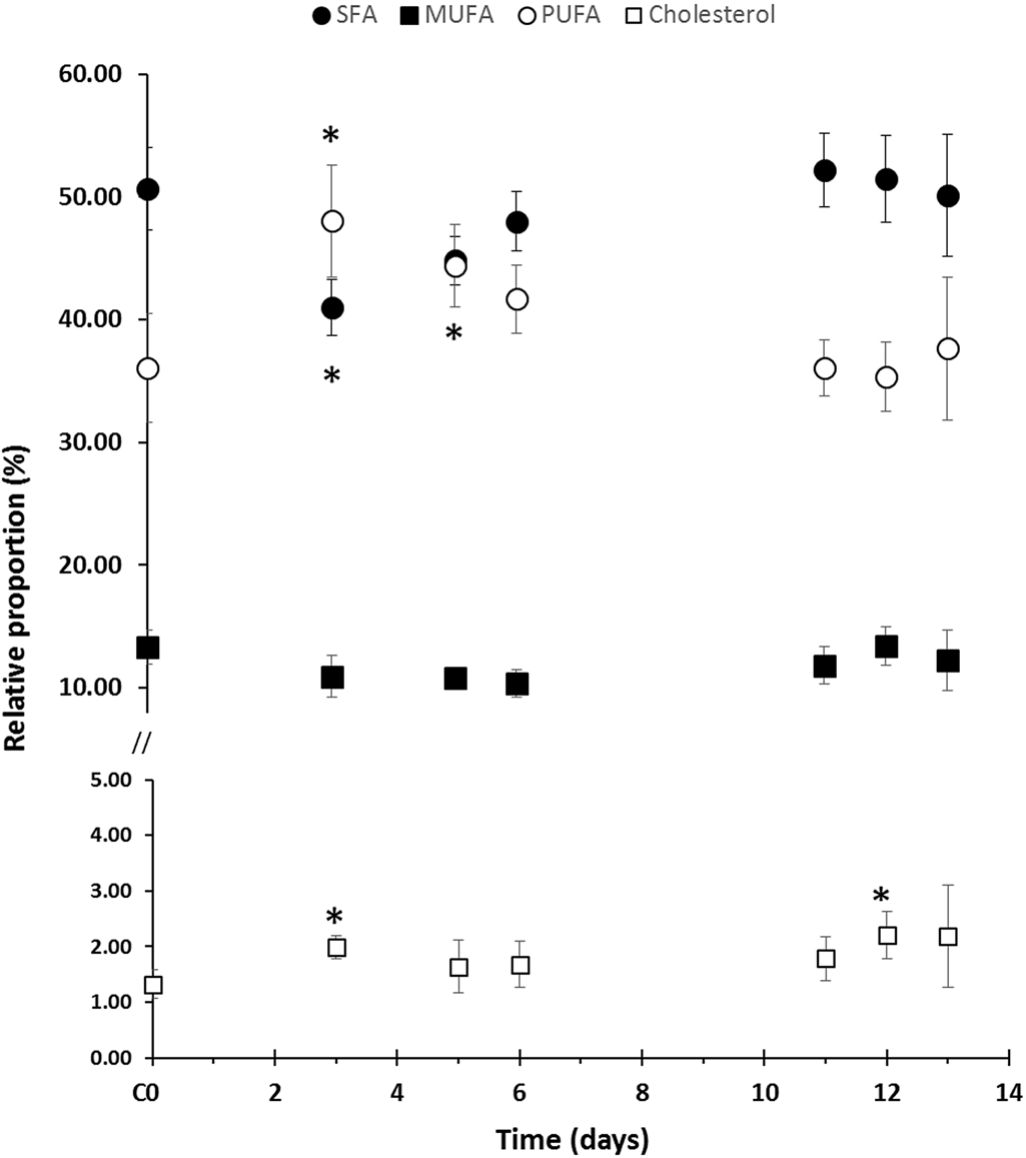
**Relative distribution of FA classes and cholesterol according to sampling time.** The C0 value represents the average of all control samples over the time series. A star (*) indicates a significant difference with the control value (*P*<0.05, Tukey's HSD ANOVA test).

Total lipid amounts were stable throughout the heat stress experiment (Fig. S2; ANOVA *P*=0.66), but some variations within each FA subclass proportion were observed (Table 1; SFA/MUFA/PUFA: d.f.=12, *F*=11.59, *P*=3.5e-15; n-3/6/9: d.f.=12, *F*=5.817, *P*=7.3 10^−8^). While the proportion of MUFA was quite similar at each time point (10.35%-13.32%), the proportions of SFA and PUFA showed significant variations (Fig. 1). The first significant variation was observed at d3 (3 days, +3°C). While SFA were the major constituents in the controls (C0) when compared to PUFA, a switch in SFA/PUFA relative proportions was noticed in stressed samples at d3 (tSFA=−9.6, *P*<0.001; tPUFA=12.0, *P*<0.001) associated with a slight increase in cholesterol proportion (t=6.34, *P*<0.001). From d5, SFA and PUFA were found in the same proportions, and both returned progressively back to their initial proportions (d11) and stayed stable until the end of the time series, even 24 h after the shift back to the initial temperature. Concerning the cholesterol, following its early increase, it returned rapidly to its initial proportion (d5). It stayed stable all along the 32°C plateau and slightly increased during the second thermal stress (d12, t=3.57, *P*=0.015) and then stabilised.

Beyond this relatively simple scheme of the thermal stress response, more complex profiles were observed in the FA composition for each subclass (SFA, MUFA, PUFA). At d3, the important proportion of PUFA is mainly attributed to the n-3 PUFA (DHA and C18:5) and at a lesser extent to the n-6 PUFA (ARA). The decrease of SFA was essentially due to the palmitic acid, C16:0. While SFA and PUFA returned to their initial proportions, the composition of these subclasses was distinct from their C0 values, with at d11 respectively more C20:0 and C22:0 less C16:0, more C20:2/C20:4/C22:2 and C22:4 and less C18:4. This new composition then remained relatively stable until the end of the time series. From d11, a decrease in SDA, one of the FA biomarkers of *Symbiodinium,* was observed.

### Lipid pathways and ROS scavengers are overexpressed during stress

**Fig. 2.**
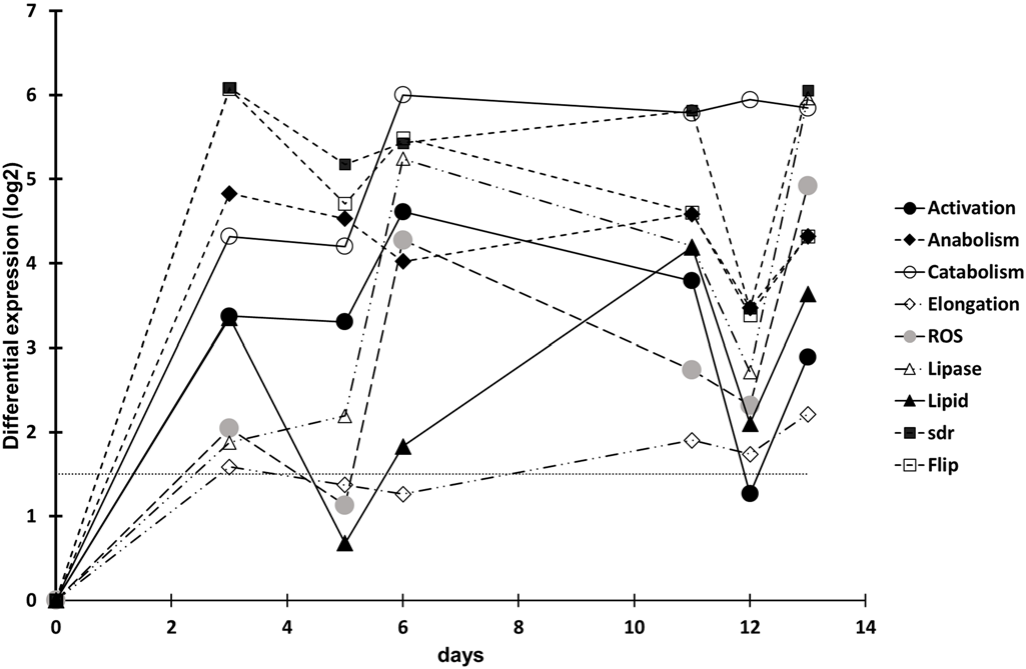
**Variation in gene expression involved in metabolism and regulation of lipids and ROS.** The dashed line indicates the threshold of significant DEG with D0.

A total of 40,221 giant clam RNA contigs were identified for which more than 50% were annotated and then clustered as mentioned in the Materials and Methods section. 473 contigs, identified as participating in the metabolism pathway of lipids or ROS scavenging, were sorted out into 9 distinct clusters. The DEG levels during the time series generated similar regulations for these clusters (Fig. 2). At the first point in the time series, all clusters exhibited a peak of upregulation, when the temperature had increased from 26°C to 29°C after 3 days of thermal stress. This point was particularly pronounced for ‘Flip’ and ‘Sdr’ clusters where genes responsible for the rearrangement of lipids in the membrane and in fatty acid modifications, such as genes encoding delta 4, 5, 6 and 9 desaturases, were affected. At d5 (31°C), upregulation decreased slightly for each cluster only to increase again at d6. During the ‘plateau’ (32°C; d6 to d11), except for the ‘ROS’ and the ‘Lipid’ clusters which respectively exhibited a down- and upregulation, the profiles of the clusters remained nearly stable. The ‘Catabolism’ and ‘Sdr’ were the highest upregulated clusters and ‘Elongation’ the lowest. At d12, when the temperature dropped back to 26°C, all the clusters, except ‘Catabolism’, experienced a down regulation. One day after the return to 26°C, the upregulation of the clusters increased again.

### The free radical scavenging ability was increased at 32°C

In addition to the expression of genes involved in ROS scavenging (‘ROS’ cluster), the apolar fraction of the samples was also tested for its free radical scavenging activity (Fig. S3). The DPPH test revealed a significant increase in their antioxidant activity when the temperature reached 32°C (after 6 and 11 days, +10.8%±2.22 and +11.16%±2.25 respectively). The unstressed samples exhibited no difference after their return to 26°C.

### *Symbiodinium* exhibited cell damage during thermal stress

**Fig. 3.**
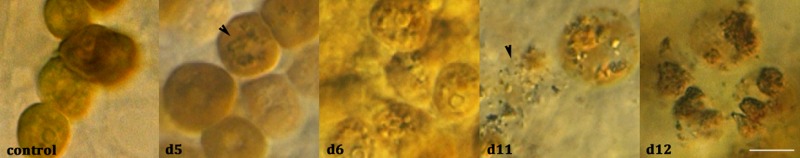
***Symbiodinium* morphology in *Tridacna maxima* course of the experiment.** *Symbiodinium* were observed by light microscopy in samples collected at d0 in control tank and at d5, d6, d11 and d12 in the heat stress experiment tank. Scale bar: 10 µm.

While the giant clam mantle remained coloured for the duration of the thermal stress experiment, the microscopic observation of the siphonal mantle samples, taken at each point of the time series, revealed morphological changes of *Symbiodinium*. With increasing temperature, *Symbiodinium* gradually exhibited cell damage (Fig. 3). While in the control, the *Symbiodinium* cells were ≤13 µm in diameter, some cells reached up to 16 µm diameter at d5, when the temperature reached 31°C and to 17.5 µm at d11, after 5 days at 32°C (Fig. 3, compare d5 and d11 *Symbiodinium* size to control). Moreover, when the temperature reached 31°C, sparse *Symbiodinium* cells (<1%) were found harbouring aggregates in their cytosol (indicated by an arrowhead in Fig. 3 d5). The frequency of these aggregates dramatically increased at 32°C with some areas composed solely of damaged *Symbiodinium* cells (Fig. 3 d6) while other areas were still composed of control-like cells. After 5 days at 32°C*,* the rupture of *Symbiodinium*, cells as well as cellular debris, were observed (indicated by an arrowhead Fig. 3 d11). At d12, when the temperature dropped to 26°C, this phenomenon was still visible and even exacerbated, with *Symbiodinium* cells exhibiting a new phenotype characterised by large dark granulations (Fig. 3 d12). No sample was observed at d13.

## DISCUSSION

### The FA composition of French Polynesian wild *T. maxima* reflects its tropical location

The lipid content at 26°C is equally composed of saturated and unsaturated FA in French Polynesian *T. maxima*, which are richer in SFA than the Egyptian *T. maxima* (Mostafa and Khalil, 2014). Similar to HVA, SFA are more abundant in organisms found in warm climates (Logue et al*.*, 2000); therefore, the higher proportion of SFA in French Polynesian giant clams might be due to the temperature of the sea water, which is warmer in French Polynesia than in Egypt (25-32°C vs 21-28°C). Similar ratios have been found in tropical corals [for review see Imbs (2013)]. Another interesting finding was the diversity of the SFA which goes from C12:0 to C22:0 in French Polynesia, instead of C14:0 to C18:0 in Egypt. The presence of C20:0 and C22:0 has rarely been observed in marine organisms. Taking into account that long chain FA abundance is a characteristic of warm environments (Brodte et al*.*, 2008; Logue et al*.*, 2000) their presence in French Polynesian giant clams, as well as in corals from the China Sea (Imbs et al., 2007), also reflects the effect of tropical location. This FA richness may also underpin their ability to adjust their FA composition to daily environmental fluctuations which can be different from organisms adapted to constant temperatures (Van Dooremalen et al*.*, 2011).

With respect to FA proportions, palmitic acid (C16:0) is the dominant FA in French Polynesian giant clams (35.17%). This accumulation could be attributed either to a high activity of biosynthesis or to low biosynthetic pathways leading away from palmitate content or even to diet. For the unsaturated FA, the PUFA are dominant in the giant clams from French Polynesia and Egypt (36.04% and 47.76% respectively). Among these PUFA, the n-3 PUFA are dominant in both regions (23.62% vs 27% in Egypt) and their global composition is similar that of other marine invertebrates, where SDA and DHA are present as major compounds (Joseph, 1982). Daily intake of n-3 PUFA is recommended by different Food Safety Agencies (Tur et al., 2012) and, as demonstrated by Mostafa and Khalil on hypercholesterolemic rats (Mostafa and Khalil, 2014), the consumption of these giant clams should have positive effects on human health.

### Early and late changes in FA composition along with gene expression during thermal stress

Thermal stress is known to have physiological effects on the organisms such as modifying membrane fluidity, inducing a rearrangement of the FA compounds (HVA), increasing ROS production, destabilising nucleic acids and proteins, and disrupting enzyme activities (Kültz, 2005; Pierce et al., 2005). Herein we showed that the FA composition of *T. maxima* changed in response to thermal stress. During the heating phase (<d6) an increase of longer carbon chain PUFA (i.e. DHA and ARA), and of cholesterol, was observed in association with a decrease of C16:0 (Table 1). With respect to FA in the membrane phospholipids, an increase in PUFAs, even those with longer carbon chains, might influence membrane fluidity and can thus have detrimental affects on organism; however, the cholesterol increase could compensate for this effect. This FA pattern could also be important for the cell, by changing the availability of lipid storage which can lead to the differential use of some FA, notably in cell signalling, energy resources, or in metabolic processes such as eicosanoid biosynthesis. Similarly, mussels and oysters have shown to increase their C20:4 in response to increases in temperature (Pernet et al., 2007a). During the plateau, the SFA/UFA ratio recovered to its initial level but SFAs and UFAs differed in their compositions when compared to the control. After 5 days, *T. maxima's* FA response to heat stress (+6°C) is mainly characterised by the increase of the proportion of longer carbon chain FAs independent of the degree of saturation [C20:0, C22:0, C20:2n-6, C22:2(7,15), ARA, C22:4n-6]. These results suggest that FAs with very long chains have an important role in the stress response of *T. maxima* to thermal stress.

The increased proportion of longer carbon chain FA required enzymatic activities such as those of desaturases, elongases and lipases (McNeill et al., 1996). The analysis of the gene expression over the time series highlighted significant differences between control and stressed samples. We first observed a systematic increase in the expression of genes involved in lipid and ROS pathways a few hours after the onset of the thermal stress (+3°C d3 versus d0, and 24 h after a −6°C stress at d12 versus d11). The gene products identified in the bilayer transfer of phospholipids in the membrane (‘Flip’ cluster), desaturases and other enzymes of FA modification (‘Sdr’ cluster) were notably upregulated. These fast and high activations could contribute to an increase in the pool of the functional enzymes responsible for the FA rearrangement and ROS scavenging. Upregulation was maintained throughout the thermal stress phase and is in accordance with the increase of the metabolic rate which follows the increase of environmental temperature in ectotherms (Brown et al., 2004; Hochachka and Somero, 2002). This constant response could compensate for the increase of protein degradation, for example due to their misfolding, and/or might reflect the enhanced protein recycling activities observed in giant clams (Ungvari et al., 2013). Alternatively, it is possible that after 6 days at 32°C, the FA response was still not complete and needed even more time to optimise the thermal acclimation response. These two hypotheses are not mutually exclusive and might explain the high level of upregulations maintained throughout the 6 days. Interestingly for all clusters, when even a brief decrease in the overexpression was observed in response to a decrease in temperature (d12), previous upregulation was recovered within 24 h of the drop (d13). Because the protein recycling activities should decrease once the initial temperature has been restored, the unexpected upregulation could be the result of the need for functions to resume to normal in order for the initial arrangement of membrane lipids to be preserved.

Considering ROS scavenging, the DPPH test showed a significant increase of antioxidant capacity during the plateau for the apolar composition and the DEG reveals an upregulation of SOD and catalases. This strongly suggests that a ROS stress occurred during the thermal stress and that ROS scavenging by the lipids as well as by the enzyme pool was strengthened.

### Two *Symbiodinium* cell death processes are induced during thermal stress

Simultaneous to the gene expression and FA composition modifications, our microscopic observations clearly detected a change in *Symbiodinium* morphology during thermal stress. These changes in *Symbiodinium* phenotype appeared when the temperature reached 31°C and increased throughout the thermal stress experiment even at the moment when the temperature returned to 26°C. Therefore, as described for *Symbiodinium* in sea anemones and corals (Dunn et al., 2004; Strychar and Sammarco, 2009), the higher the temperature, the more damaged *Symbiodinium* are. In accordance with these observations, a decrease of C18:4n-3, a *Symbiodinium* biomarker, was observed until d11, at the end of the plateau. The fact that other *Symbiodinium* biomarkers such as DHA or EPA (C20:5 n-3) exhibited a weak or no tendency to decrease might be explained by the ability of molluscs, due to the presence of appropriate enzymes, to perform PUFA biosynthesis (Monroig et al., 2013). It has been shown that the Pacific oyster *Crassostrea gigas* is able to produce DHA or EPA when PUFA are not provided through algae feeding (Waldock and Holland, 1984), and this might reflect a constant need for EPA and DHA in those species.

Two different *Symbiodinium* morphological hallmarks were observed. One consisted of an increase in *Symbiodinium* volume (d5, 31°C) with the appearance of small aggregates in *Symbiodinium* cells (d6, 32°C), leading to the complete rupture of the cell (d11). The second appeared later (d12) and was characterised by large dark granulations. The former phenotype strongly suggested necrosis modalities while the latter could correspond to apoptosis (Kroemer et al., 2009). Together, these observations strongly suggest that for *T. maxima*, as in sea anemones and corals (Dunn et al., 2004; Strychar and Sammarco, 2009), there are at least two *Symbiodinium* cell death processes which occur in the host upon thermal stress. *Symbiodinium* are live and exosymbiont in a digestive tubular system which communicates with the stomach in *T. maxima* (Norton et al., 1992). It has been proposed, as in some nudibranchs, (Burghardt and Wägele, 2014; Norton et al., 1992; Soo and Todd, 2014), that *Symbiodinium* might be farmed to supply nutrients as required. The necrosis-like degradation of *Symbiodinium* observed during the increase in temperature might offer to *T. maxima* an important and rapid source of food which could delay the lipid storage depletion (total lipid proportion is stable along the thermal stress). Consistent with the energy-budget model (Anthony et al., 2009; Brown et al., 2004), this could delay the onset of their mortality.

## MATERIALS AND METHODS

### Experiment

Seventy specimens of *T. maxima* from 5 to 7 cm in length were sampled during the cold season (September 2012) in Tahiti lagoon (authorisation N° 1582 in the official journal of French Polynesia, 23rd February 2012, 17°42′13.64″ South – 149°35′8.99″ West) and were placed in open circuit aquaria for acclimation for 15 days. The experiment was performed in aquaria with running seawater (100-litre tanks), with a water renewal rate of 24 litres per hour. Food was supplied exclusively through seawater renewal. Two control tanks were maintained at ambient temperature (26°C) throughout the experiment, while the two other tanks were used for thermal stress. A header tank containing a heating resistor and a thermostat IC 901 (Eliwell France) provided warm seawater to the stressed tanks while mimicking the daily fluctuations (Fig. S1). A circulation pump (EHEIM Compact 600/150) adjusted at 600 L/h, a digital thermometer and a probe (Hobo) for continuous temperature and light monitoring (one measure every 10 min) were placed in each tank. The experiment was performed outdoors, under transparent sheets, and light was compensated in order to get a luminosity comparable to the one observed in the lagoon, measuring between 5500 and 18,000 lux as a function of the time of the day and the climate conditions.

### Animals

Giant clams were divided into two groups. One group (*n*=35) was maintained at the lagoon temperature, 26°C (control group), and the second group (*n*=35) was subjected to a gradual temperature increase of 1°C/day, until they reached 32°C. The temperature was maintained at 32°C for 5 days, then reduced to 26°C over the course of a day, and held at this temperature for an additional day. Samples were collected at days 3, 5, 6, 11, 12 and 13 (Fig. S1).

Five giant clams from each tank, representing a sample from each of the collection points, were processed one after the other, quickly killed with a scalpel and dissected to collect mantle pieces. As our study focused on mRNA, fatty acids and the relative cholesterol composition of the mantle, two pieces of mantle were collected: one piece (2/3 of the mantle) was immediately immersed in liquid nitrogen, kept for 24 h at −80°C, lyophilised, weighed and transported to the laboratory at ambient temperature for lipids extraction (107,2–549,9 mg). The second piece (1 cm²) was conserved overnight at 4°C in 0.5 ml of RNAlater solution (Ambion) and then conserved at −80°C until RNA extraction.

### Lipid extraction

Each sample was subjected to three successive lipid extractions, and the three extractions were then combined, following the protocol developed by F. Mohamadi (F. Mohamadi, PhD thesis, University of Perpignan, 2014). Samples were sonicated for 10 min first with a methanol: water (4/1 v/v) mixture, and again with a mixture of dichloromethane: water (2,5/2 v/v). All extracts were collected successively and centrifuged together at 1920 ***g*** (3500 rpm) for 10 min. The lipidic apolar phase was evaporated under a nitrogen stream, weighted and kept at −20°C (6,6–18,2 mg).

### Sample preparation

A small part of the apolar fraction was then derivatised prior to conducting gas chromatographic analyses according to the method described by Xu et al*.* (2009). Derivatisation was usually performed before GC/MS analysis. This process improves sample volatility, stability, sensitivity and selectivity (Xu et al*.*, 2010).

A 1.5 ml of methanol, 0.2 ml of toluene and 0.3 ml of an 8% (methanol /HCl 85/15, v/v) solution were added to the lipid samples. After 15 min in the ultrasonic bath, the samples were then heated for 16 h at 45°C. Fatty acid methyl esters (FAME) and sterols were extracted twice with hexane/dichloromethane (4/1, v/v). The supernatant was transferred to a 5 ml glass vial and dried by nitrogen desiccation. Then, 0.1 ml of Bis(trimethylsilyl)trifluoroacetamide (BSTFA) and 0.1 ml of pyridine were added to the residue for silylation derivatisation at 45°C for 16 h. The derivatives were then centrifuged for 5 min at 1920 ***g*** and the supernatants were transferred into a vial.

Methylated derivatives of standard fatty acids corresponding to decanoic acid (C10:0), dodecanoic acid (C12:0), myristic acid (C14:0), PAM: palmitic acid (C16:0), STA: stearic acid (C18:0), OLA: oleic acid (C18:1 n-9), eicosanoic acid (C20:0), docosanoic acid (C22:0), tetradecanoic acid (C24:0) and trimethylsilyl derivative of commercial cholesterol were prepared in a similar way.

### Lipid analysis

Analysis of the lipid apolar fraction and derivative standards were carried out on a Focus GC coupled with a GC-MS Thermo Focus DSQII equipped with a AI 3000 II injector (Thermo Fisher Scientific France). The GC was fitted with a Supelco SPB-50 capillary column (30 m×0.25 mm×0.25 µm) (Sigma-Aldrich). Helium was used as the carrier gas at 1 ml/min. The GC oven temperature was as follows: 150°C for 3.5 min, then 20°C/min to 200°C, held for 10 min, and finally 3°C/min to 280°C, held for 10 min. 250°C and 290°C were set as the ion source and the transfer line temperatures respectively. The transfer line temperature was operated in electron impact (EI) mode (70eV). Data acquisition was performed in full scan mode from *m/z* 40 to 600.

Compounds were identified by comparing mass spectra and retention data with standard values, and those available in libraries (NIST 2.0), through the use of characteristic m/z values (Härtig, 2008) and their equivalent chain lengths (ECL) (Mjøs, 2006). The relative composition of the identified compounds was estimated using peak areas and expressed in percentage.

### *Symbiodinium* observation

Pieces of the siphonal mantle of two giant clams per point on the time series and for each experimental condition were fixed and observed in light microscopy with a Zeiss microscope (Axio Imager M2). The protocol we used was adapted from the method developed by Berteaux-Lecellier et al*.* (1995). In brief, tissues were immediately fixed in a 7.4% paraformaldehyde solution. They were rinsed in PBS X1 (PBS X10: Na2HPO4: 0.8M; NaH2PO4, 2 H2O: 0.2M; NaN3: 0.5%) then crushed between a polylysinated microscope slide and a cover-glass with a needle. The material was rinsed twice with PBS 0.05%. After a final rinse with distillated water, samples were prepared on a blade with a drop of mounting solution and preserved at −20°C.

### Gene expression

RNA from each sample was extracted following the Trizol Reagent manufacturer protocol (Invitrogen, Cat. No. 15596-018) with a modification of the homogenisation step: samples were rinsed with 0.5 ml of PBS 10X, dissected in a refrigerated petri box, with a scalpel, and transferred to a 2 ml vial containing 0.5 ml of TriZol. Three freeze/thaw cycles were conducted and samples were placed at 4°C overnight. Separation, precipitation and washing steps were then conducted. The integrity and quality of total RNA was assessed using a Bioanalyser (Agilent Technology). Only samples showing high quality RNA (RNA Integrity number >8) were used for RNA-seq analysis.

For each point in the time series, the 5 RNA samples were pooled before sequencing. Sequencing was conducted by Genoscreen (Lille, France) using a shotgun 2×100 bp (454) on HiSeq 2500, with a read depth comprised between 1.5-2.5 Gb sequences per sample. The raw data are available on Bioproject, the NCBI BioProject database (http://www.ncbi.nlm.nih.gov/bioproject, accession PRJNA309928) (PRJNA309928). Only reads with a Q>30 were conserved and assembled by TrinityRnaseq software (Haas et al*.*, 2013). Resulting contigs with an open reading frame >30 amino acids were selected and those blasting with *Symbiodinium* databases with a threshold of 10^−5^ were discarded. Resulting contigs were blasted (rpsblast) for annotation with the CDD database (ftp://ftp.ncbi.nih.gov/pub/mmdb/cdd/, release 02/14/2014) of NCBI, with a threshold of 10^−15^. The annotated contigs, involved in metabolism and management of the lipids and ROS scavenging, were selected. Their functions were confirmed by blasting them with the NCBI protein database with the same threshold (top hits in Table S1) as we simultaneously checked that no *Symbiodinium* genes were detected. After this selection scheme, contigs were sorted into 9 distinct clusters. The ‘Activation’ cluster consists of acyl-coA synthetases (fatty acids-facs, medium chain-macs and very long chain vl-facs genes), fatty acid coA ligases and regulatory binding elements such as srebp genes. The ‘Anabolism FA’ cluster consists of genes implied in the anabolism of fatty acids (2-enoyl thioester reductases, acetyl-coa carboxylases, acyl-acp thioesterase). The ‘Catabolism’ cluster consists of genes implied in catabolic reactions of fatty acids (3-hydroxyacyl-coa dehydrogenase, crotonases). The ‘Elongation’ cluster consists of enzymes specifically implied in the elongation of the chain of fatty acids (gns1/sur4 family). The cluster ‘ROS’ was built with the glutathione, SOD and catalase gene families. For the management of lipids, other desaturases (such as delta4, 5, 6, 9 desaturases), dehydrogenases, and reductases were associated in the cluster ‘Sdr’; phospholipases, thioester hydrolases, esterases and triglyceride lipases in the cluster ‘Lipase’; and flippases, scamblases, lipocalins and acyltransferases in the cluster ‘Flip’. Stard genes and phosphotransferases, implied in the transfer of phospholipids between the membranes, were grouped in the cluster ‘Lipid’.

The differentially expressed gene (DEG) analysis was conducted by the Trinityrnaseq pipeline (Haas et al., 2013), with fragment coverage of 100 bp and Bowtie 2 software (Langmead and Salzberg, 2012) on the annotated contigs. The normalised estimation of the DEG levels in function of this coverage (HMM-FKPM) was conducted with RSEM software (Li and Dewey, 2011) and EdgeR package (Robinson et al., 2010). The DEG levels of the contigs, expressed in log2, were evaluated by comparing their expression levels between heated and control tanks per time point. For each contig, the values were then adjusted according to the first time point (d0). Finally, for each cluster, the maximum value of all contigs per cluster was retained per time point.

### Antioxidant activity

The antioxidant activity was estimated with a DPPH free radical test following the Brand-Williams method (Brand-Williams et al., 1995) with some modifications: 20 µl of lipid extract was added to 200 µl of a 2.2-diphenyl-1-picrylhydrazyl (Aldrich^®^ 1898-66-4) in methanol (0.2 mM); 96-well microplates were used. The incubation was performed at ambient temperature without light. The absorbance was measured every minute at 515 nm using a microplaque lecturer Aviso, Sirius HT (Ebersberg, Germany). In order to determine the optimal time to measure with the spectrophotometer, a kinetic curve was first established for different concentrations (raw sample and samples diluted 10 and 100 times) over 240 min. All measures were conducted in triplicates. After 100 min from the first measure (initial time: i), a bending of the decrease of absorbance was observed and this time was selected as a final time (f). The net decrease of absorbance [ΔAbs=Abs final (f) – Abs initial (i)] per sample was evaluated by subtracting from this decrease (sample with DPPH), the decrease of absorbance of the sample with only MeOH and the decrease of absorbance of DPPH in MeOH during the same time, following the formula: ΔAbs=(Abs_f_–Abs_i_)_sample+DPPH_–(Abs_f_–Abs_i_)_sample+MeOH_–(Abs_f_–Abs_i_)_MeOH+DPPH_. The corresponding 100% decrease of absorbance of DPPH was evaluated as: Δ100%Abs=–(Abs_i_)_sample+DPPH_–(Abs_f_–Abs_i_)_sample+MeOH_–(Abs_f_–Abs_i_)_MeOH+DPPH_. The radical scavenging activity (RSA %) was evaluated by the ratio between the net decrease of absorbance and the expected value with total captured DPPH: RSA %=ΔAbs/Δ100%Abs×100.

### Statistical analysis

Data are presented as the means±standard deviations. Data were statistically processed using a MANOVA analysis to determine significant differences between the groups. The effects of temperature were statistically analysed by a one-way ANOVA. Differences between treatments were tested using Tukey's HSD ANOVA procedure, correcting for family-wise error-rate for multiple pairwise comparisons. All procedures were carried out using R 3.2.
